# Psychometric Properties of the Arabic Version of the Behavioral Intention to Interact With Peers With Intellectual Disability Scale

**DOI:** 10.3389/fpsyg.2020.01212

**Published:** 2020-06-19

**Authors:** Ghaleb H. Alnahdi, Susanne Schwab

**Affiliations:** ^1^Department of Special Education, College of Education, Prince Sattam bin Abdulaziz University, Alkharj, Saudi Arabia; ^2^Center for Teacher Education, University of Vienna, Vienna, Austria; ^3^Optentia Research Focus Area, North-West University, Vanderbijlpark, South Africa

**Keywords:** BIS, psychometric properties, attitudes, intellectual disability, inclusive education

## Abstract

According to literature, students’ attitudes toward peers with disabilities are crucial for the social inclusion of students with disabilities. Therefore, knowledge about students’ behavioral intention to interact with peers with intellectual disability (ID) can help improve the social inclusion of students with ID. This study aimed to examine the psychometric properties of the Arabic version of the Behavioral Intention to Interact with Peers with Intellectual Disability Scale (BIS). Data were collected from 887 elementary school students (591 girls and 296 boys) from third to sixth grades in Saudi Arabia. Psychometric properties of the BIS were examined with a confirmatory factor analysis, measurement invariance analysis (across gender), and reliability scales (internal consistency). Good indicators were obtained for the construct and the convergent validity of the BIS. The results supported the two-dimensional structure of the BIS. The internal consistency of the BIS and each of its subscales was good. Furthermore, no measurement variance was found for boys and girls students. The Arabic version of the scale showed good psychometric properties and therefore can be recommended to measure students’ behavioral intention to interact with peers with intellectual disability.

## Introduction

Inclusive education has started to evolve around the world. Saudi Arabia is not an exception. In the last two decades, there was a clear increase in the inclusion of students with disabilities—including students with intellectual disabilities (ID)—in regular schools (Alnahdi et al., 2019a). However, the placement of students with disabilities in schools does not guarantee inclusion in the sense of overcoming barriers to social participation. According to literature, students with disabilities, especially those with ID, are at high risk of being socially excluded. They have less interactions with their peers in school, less friends, are more often rejected, experiencing lower levels of social inclusion. For an overview, see recent studies on social participation of students with special needs (Koster et al., 2010; Bossaert et al., 2013; Schwab, 2018b). For instance, Garrote (2017) demonstrated that of 43 students with ID, 19 students were rejected and/or isolated and only one student with ID was popular.

Even if the barriers limiting the social inclusion of students with disabilities are complex and not fully understood, several authors have outlined students’ attitudes toward peers with disabilities as a crucial influence factor (Hellmich and Loper, 2018; Schwab, 2018a).

### Attitudes Toward Peers With ID

The theory of planned behavior provides a theoretical basis implying that attitudes shape behavioral intentions and behavior (Ajzen and Fishbein, 1977). Another theoretical foundation is the theory of cognitive dissonance, which assumes that people have an inner drive to keep attitudes and behavior in balance (Festinger, 1957). One of the most cited models of attitudes—the ABC model (Triandis, 1971)—includes a behavioral component. The ABC model refers to A as the affective component (feelings about the object), B as the behavioral component (behavioral intentions), and C as the cognitive component (beliefs; Eagly and Chaiken, 1998). According to recent literature reviews, the kind of disability someone has is important when investigating students’ attitudes toward peers with disabilities (De Boer et al., 2012; Schwab, 2018a). While students tend to hold a rather positive attitude toward peers with learning disabilities or physical disabilities, they have a more negative attitude toward peers with ID. Moreover, these reviews clearly indicated a more positive attitude toward peers with disabilities by female students compared to male students. Georgiadi et al. (2012) suggested that girls showed a more positive attitude toward peers with ID than did boys. Unfortunately, it is unclear whether this group difference might be biased by measurement variance, as most of the studies did not check if the instrument used shows measurement invariance between these groups. As “the same attribute must relate to the same set of observations in the same way in each group,” it is problematic to compare sum scores without showing measurement invariance beforehand (Borsboom, 2006).

Attitudes toward people with ID are an important aspect in ensuring that schools will be a welcoming environment for students with ID. Therefore, it is important for professionals and researchers to have assessments/measures to examine and study students’ attitudes toward peers with disabilities.

The behavioral intentions of students to interact with peers with ID are essential in inclusive education. These attitudes play an important role in determining whether students are willing to interact with peers with ID, both in and outside of school. Up to now, most research on inclusive education has focused on social participation during school time. Therefore, there is a gap in students’ social interactions with peers out of school. Using reliable scales to measure children’s intention to interact with peers with ID is an essential step in understanding students’ attitudes toward peers with ID. This will further allow the measurement of changes as a result of interventions to encourage interaction with peers with ID. For the Arab region, a need for scales that meet high psychometric standards has been noted, especially within the context of inclusive education (Suleiman and Bablawi, 2011). One internationally used scale to assess students’ behavioral intentions is the Behavioral Intention to Interact with Peers with Intellectual Disability Scale (BIS; Siperstein et al., 2007). This scale has already been used in different samples from different countries and regions, such as the United States (Siperstein et al., 2007; Brown et al., 2011) and Canada (Siperstein et al., 2007). However, no data are available for Saudi Arabia (or any other Arabian country). Brown et al. (2011) showed in their study that, as found for overall attitudes scales, students’ behavioral intentions toward peers with ID were more negative compared to their behavioral intentions toward peers with physical disabilities. Likewise, Schwab et al. (2016) showed that students are less likely to sit next to a “new” virtual classmate with Down syndrome than a student with no obvious disability. Furthermore, Gasser et al. (2012) analyzed attitudes toward people with ID using an experimental study in which students decided if a protagonist would include a child with ID in different kinds of group activities. The results of their study confirmed that the intention to include a student with ID in school or social group activities is lower compared to the intention to include a child with physical disabilities.

### The Present Study

As mentioned earlier, there are several gaps in the literature. First, the psychometric properties of the Arabic version of the BIS (BIS-AR) have not yet been evaluated. Therefore, this study investigated the factorial structure as well as the reliability of the BIS-AR. Moreover, as research on gender differences in students’ attitudes after checking for measurement invariance is limited, this study provided information on whether comparison of total scores is allowed by investigating measurement invariance between girls and boys.

## Materials and Methods

### Sample

Students’ data sets were collected from a convenience sample of elementary schools in a region of Riyadh in two cities (Riyadh and Alkharj). This study was approved by the institutional review board (IRB) of the university and guidelines were followed to ensure anonymity and confidentiality of participants and parents’ responses. In total, 887 students answered the paper-pencil questionnaire. The sample consisted of 67% girls and 33% boys from third to sixth grade; 26% were in the third grade, 18% were in the fourth grade, 35% were in the fifth grade, and 20% were in the sixth grade. Boys were representing 16% of the sample in the third grade, 38% of the sample in the fourth grade, 41% of the sample in the fifth grade, and 38% of the sample in the sixth grade. The students’ age range from 8 to 14 years old (*M* = 10.4, SD = 1.24).

### Measures

The BIS was used to assess students’ behavioral intentions. This scale consists of 12 items, which can be divided into two subscales (Siperstein et al., 2007). While the first six items refer to behavioral intentions in school (e.g., “Work with a student with ID on a project in class”), items 7–12 refer to behavioral intentions outside of school (e.g., “Invite a student with ID to your home”). Items are answered on a four-point Likert scale. All items have been worded positively. A higher score indicated higher behavioral intention to interact with peers with ID. For the original version (Siperstein et al., 2007), acceptable internal consistency was shown for the total scale (Cronbach’s α = 0.932), as well as for both subscales (in school: Cronbach’s α = 0.872; out of school: Cronbach’s α = 0.872). Based on the author’s literature review, no study examined the two-dimensional factor structure of the BIS using CFA.

After we obtained the permission from Prof. Gary Siperstein from University of Massachusetts Boston to translate and validate the Arabic version of the scale, the translation of the BIS from English to Arabic was done following the recommended procedures for cross-cultural adaptation of scales (Beaton et al., 2005). Two bilingual (Arabic and English speaking) researchers with doctoral degrees in education translated the original English version to Arabic. Then, the two new Arabic versions were compared and were combined into a single Arabic version. After that, the Arabic version was sent to another bilingual researcher with no previous knowledge about the English version of the scale for translation back to English. In the next step, the original English version of the scale was compared with the back-translated English version and a slight change was made to the Arabic version based on this meeting to ensure the meaning was preserved in the Arabic version. Finally, the Arabic version was pilot tested with a sample of 53 students to ensure that the Arabic version had good internal consistency (total scale: Cronbach’s α = 0.925; in-school subscale: Cronbach’s α = 0.811; out-school subscale: Cronbach’s α = 0.932), and to ensure that all items were clear and understandable to students.

In addition, we used the short version of the Chedoke–McMaster Attitudes toward Children with Handicaps scale (CATCH; Rosenbaum et al., 1986; Schwab, 2018a; for the Arabic version see Alnahdi et al., 2019b) to examine the convergent validity of the BIS. The short version of CATCH includes four items in the affective and behavioral components of attitude. Schwab (2018a) showed acceptable reliability of the short scale for primary school students (Cronbach’s α = 0.73). Moreover, very good reliability indicators were obtained for the Arabic version (Cronbach’s α = 89; Alnahdi et al., 2019b).

## Results

A confirmatory factor analysis (CFA) and measurement in variance analysis were conducted using Amos 20 software. Tests of reliability and other descriptive statistics were conducted using SPSS 25.

### Confirmatory Factor Analysis

**FIGURE 1:**
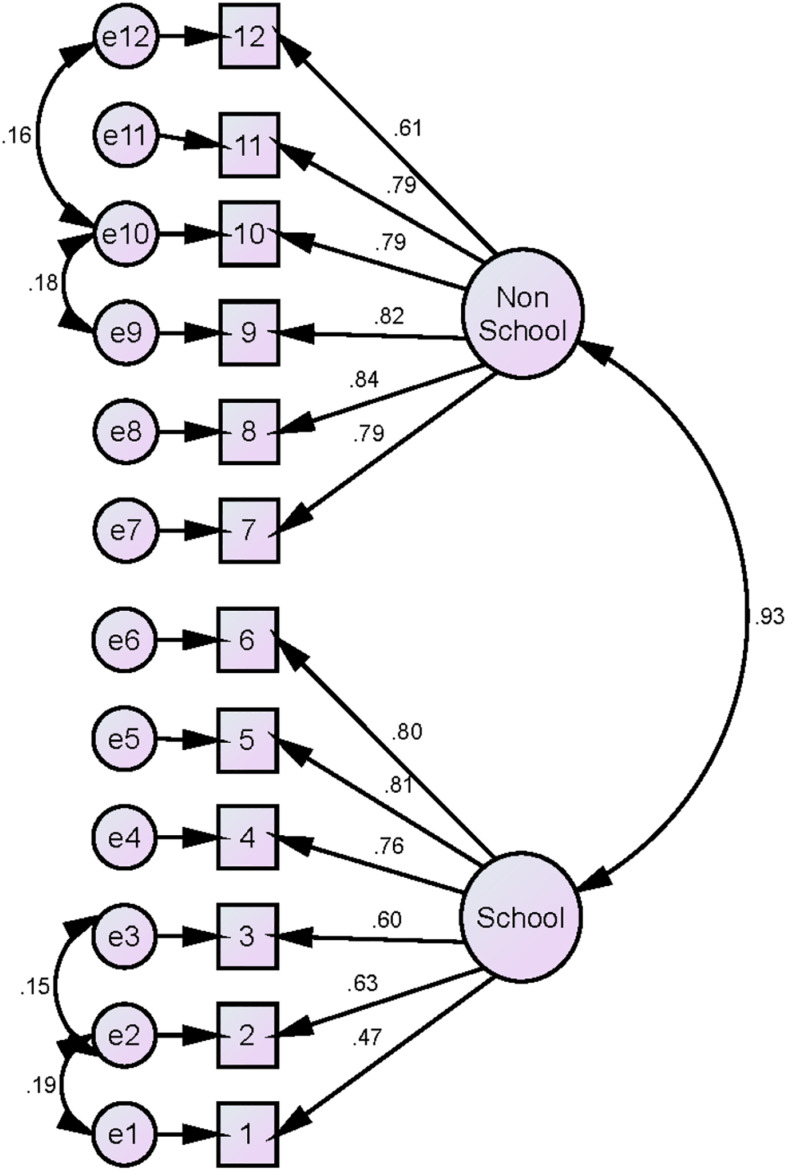
Confirmatory factor analysis of the BIS.

A CFA was conducted to examine the hypothesized two-factor structure (see Figure 1). A chi-square (χ^2^) was conducted but not discussed because of its sensitivity to large sample size, even with good fit data (Byrne, 2010). In addition, four fit indices were reported to examine whether the observed data fit the model, and considering the following value as indicator for acceptable fit (see Table 1): the Tucker–Lewis index (TLI) > 0.90 (Bentler and Bonett, 1980), the comparative fit index (CFI) > 0.90 (Bentler and Bonett, 1980; Pugesek et al., 2003), the root mean square error of approximation (RMSEA) < 0.08, and the standardized root mean square residual (SRMR) < 0.06 (Schermelleh-Engel et al., 2003). Results showed that the data fit the model with acceptable fit indices. For instance, CFI was 0.96 and SRMR was 0.042, which indicated a good fit. As shown in Figure 1, errors from items 1, 2, and 3 covaried together. Also, errors from items 9, 10, and 12 covaried together. In addition, this two factor model showed better fit indices than one-factor model or second-order CFA (see Table 2 and Appendix 1), which suggested that the two factor model is a good representation of the BIS construct to what can be provided by one-factor model or second-order CFA.

**TABLE 1:** Configural invariance of the CFA model of BIS across gender.

Model	χ^2^	df	*p*	CFI	TLI	SRMR	RMSEA	90% CI for RMSEA	
								LL	UL
Two-factors model	300.026	49	<0.001	0.960	0.946	0.042	0.076	0.068	0.084
One-factor model	371.486	50	<0.001	0.949	0.933	0.046	0.085	0.077	0.093
2nd order	371.486	50	<0.001	0.949	0.933	0.046	0.085	0.077	0.093
Gender	142.90	49	<0.001	0.946	0.928	0.051	0.081	0.066	0.096
	245.41	49	<0.001	0.957	0.942	0.044	0.082	0.072	0.093

*CFI, comparative fit index; TLI, Tucker–Lewis index; SRMR, standardized root mean square residual; RMSEA, root mean square error of approximation; CI, confidence interval; LL, lower limit; UL, upper limit.*

**TABLE 2:** Mean and standard deviations for all questionnaire items.

Item		M*	*N*	Std. deviation	Percentage agreement
1	Lend a student with ID a pencil or pen	2.54	886	0.909	88
2	Stand next to a student with ID while waiting in line	2.32	886	0.988	83
3	Go up to a student with ID and say hello	2.51	886	0.890	87
4	Talk to a student with ID during free time or lunch	2.22	883	1.045	77
5	Choose a student with ID to be on your team in gym class	2.07	884	1.106	73
6	Work with a student with ID on a project in class	2.09	885	1.090	73

**Interact in school subscale**		**13.74^a^**			

7	Sit next to a student with ID on the bus for a field trip	2.14	880	1.102	74
8	Spend time with a student with ID outside of school	1.89	885	1.149	66
9	Invite a student with ID to go out with you and your friends	2.01	883	1.128	70
10	Invite a student with ID to your home	1.89	885	1.167	66
11	Go to the movies with a student with ID	1.79	886	1.195	63
12	Talk about personal things with a student with ID	1.42	884	1.254	48

**Interact out school subscale**		**11.34^a^**			

ID, intellectual disabilities; *, highest possible score is 3; a, sum of means score; Percentage agreement, combined *yes* and *probably yes* responses.

### Convergent Validity

The convergent validity for the Arabic version of the BIS was examined by calculating its correlation with the short version of the CATCH (Rosenbaum et al., 1986; Schwab, 2018a; for the Arabic version see Alnahdi et al., 2019b). The correlation statistics showed a significant positive correlation between the Arabic version of the BIS and the CATCH (*p* < 0.001, *r* = 0.469), which can be considered a good indicator of convergent validity.

### Reliability

The reliability of the BIS and the two subscales was examined in this study sample using Cronbach’s alpha coefficients, which indicated high internal consistency: total scale α = 0.928; in-school subscale, α = 0.905; outside school subscale, α = 0.861 (George and Mallery, 2003). In addition, the average inter-item correlation for all 12 items on the total scale was acceptable (*r* = 0.508); all 66 pairs of correlations were significant (*p* < 0.01).

### Descriptive Statistics

Table 2 shows that means scores on items ranged from 1.42 (SD = 1.25) for item 12–2.54 (SD = 0.909) for item 1. Based on the theory underpinning the BIS, the range of the scores in this sample indicated positive behavioral intentions to interact with peers with ID. The total scale mean = 1.98 (SD = 0.850) (or 23.76 out of 36 as the sum of the means of all items) and the means of the subscales are 2.29 (SD = 0.765) (13.74 out of 18) for behavioral intentions to interact with peers with ID in school and 1.85 (SD = 0.958) (11.34 out of 18) for behavioral intentions to interact with peers with ID out of school. A *t*-test for dependent samples showed that the behavioral intentions to interact with peers with ID in school is significantly higher compared to the behavioral intentions to interact with peers with ID out of school (*t*(886) = 21.24, *p* < 0.01), which indicates that students expressed more willingness to interact with peers with ID in school than out of school. An independent *t*-test showed that girls expressed significantly more positive intentions to interact with peers with ID compared to boys (girls: *M* = 2.17, SD = 0.777; boys: *M* = 1.86, SD = 0.836; *t*(885) = –5.506, *p* < 0.01, Cohen’s *d* = 0.39).

### Measurement Invariance Analysis by Gender

To ensure that the BIS scale has the same psychometric properties across both genders in this study, a measurement invariance analysis was conducted. First, we tested the configural invariance by conducting CFA separately for each group (boys and girls) as suggested by Dimitrov (2010). Obtaining configural invariance refers to invariance of the model configuration across boys and girls (Mokhtari et al., 2008). Good fit indices were obtained for both genders: TLI > 0.90 and SRMR < 0.06 (Hu and Bentler, 1999; Schermelleh-Engel et al., 2003). This indicates that the BIS preserved the hypothesized two-factor structure across both genders of participants.

After the configural invariance was established, a factorial invariance analysis was conducted (Dimitrov, 2010). Chi-square difference test (Δχ^2^) was used to compare nested models with non-significant Δχ^2^ indicating invariance. Table 3 shows that metric invariance (invariant factor loadings) was not established across both genders. In addition, a scalar invariance (invariant factor loadings and invariant intercepts) was confirmed. By confirming both metric invariance and scalar invariance, a strong measurement invariance was established across both genders (Dimitrov, 2010).

**TABLE 3:** Testing for measurement invariance of the BIS across gender.

Model	χ^2^	df	Comparison	Δχ^2^	Δ df	CFI	ΔCFI^a^	RMSEA
M0	387.965	98				0.954		0.058
M1	394.943	108	M1–M0	**6.978**	10	0.955	**−0.001**	0.055
M2	396.869	110	M2–M1	**1.926**	2	0.955	**−0.00**	0.054
M3	658.130	122	M3–M2	261.261*	12	0.915	−0.040	0.071

χ^2^,chi-square fit statistic; CFI, comparative fit index; RMSEA, root mean square error of approximation; M0, baseline model (no invariance imposed); M1 (metric invariance), invariant factor loadings; M2 (scalar invariance), invariant factor loadings and invariant intercepts; M2 (scalar invariance), invariant factor loadings and invariant intercepts; M3 (invariance of item uniqueness), invariant factor loadings, item intercepts, and residual item variances/covariances; a, an indicator of invariance based on the rule for rejection of invariance (ΔCFI < –0.01; Dimitrov, 2010); bold, an indicator for invariance, *, *p* < 0.05.

## Discussion

Students’ positive attitudes toward peers with ID are an important factor for the social inclusion of students with ID. A starting point to investigate this topic is to have a reliable measurement tool. Therefore, this study examined the factorial structure and the reliability of the BIS-AR using a sample of students from different grades.

The hypothesized two-factor structure of the BIS was confirmed by the results of the CFA for the BIS-AR (Siperstein et al., 2007). Slight adjustments were made by covarying four item errors to improve the data fit. These modifications slightly improved the fit; however, the data had an acceptable fit before these modifications—that is, CFI = 0.946; TLI = 0.933—with no modifications. However, because these items had theoretically common themes, we decided to include the correlation of the errors of these items for an increased model fit.

In addition to the factorial structure, the reliability of the BIS-AR was investigated. In the present sample, the reliability statistics showed that the BIS-AR total scale and subscales have good internal consistency. Similar to the study by Siperstein et al. (2007), the Cronbach’s alpha was high for the overall BIS-AR score and was close to 0.93.

Interpreting the descriptive scores generally, it can be concluded that students have relatively high intentions to interact with peers with ID, as their answers were all close to “rather yes.” However, the more intensive the interaction gets, the less likely students are to get into interactions with peers with ID. For instance, the score for lending a pencil or pen was higher compared to spending time together (e.g., working together on a school project or be on the same team in gym class). The lowest mean score was found for sharing personal information (“Talk about personal things with a student with ID”).

Huskin et al. (2017) also showed increasing social distance by closer social interactions. Their results indicated that about 30% of undergraduates would avoid being a neighbor or co-worker to a person with mental illness. Moreover, students had a higher behavioral intention to interact with a peer with ID in school compared with interactions outside of school. This result is possibly linked with the students’ social and moral understanding. Gasser et al. (2012) showed that if students needs to decide between a peer with and without hearing impairments students think that around 50% of their peers would select the peer with hearing impairments. And they substantiated this effect because of moral reasons. Further, the results of Gasser et al. (2012) showed, students are sensitive to the situational context (e.g., school time vs. spare time). Their results indicated that students’ with hearing impairment are expected to be more often selected as a working partner in spare time compared to school time.

In addition, measurement invariance between girls and boys was tested in this study to ensure that the structure validity of the scale does not differ significantly based on gender. For the BIS-AR, measurement invariance was confirmed based on gender, which allows us to compare the total score of girls’ and boys’ behavioral intentions. In line with several other studies focusing on students’ attitudes, this comparison indicated that girls have a higher intention to interact with peers with ID than boys (Schwab, 2018a).

## Conclusion

Based on the assumption that students’ attitudes—for example, their behavioral intentions—are influencing social participation, further research needs to investigate what can influence students’ behavioral intentions. For instance, a study by Luttropp and Granlund (2010) showed that teachers’ instructions have an influence on students’ interactions. In structured activities, there may be more interaction between students with and without ID. Moreover, the study of Schwab et al. (2016) showed that peer feedback as well as teacher feedback on a fictional student with ID influenced the social acceptance of this student. Results of a meta-analysis done by Chae et al. (2018) showed that in general, interventions on students’ attitudes toward peers with disabilities are more effective if they are school-based; contact-based interventions seem to be especially effective.

In conclusion, this study found that the BIS-AR is a reliable instrument for the assessment of students’ behavioral intentions to interact with peers with ID. Therefore, this scale can be recommended for use in future studies. However, the next step would be to use the instrument in intervention studies and to investigate the practical implications of students’ behavioral intentions and its association with their real behavior in different contexts.

## Data Availability Statement

The raw data supporting the conclusions of this article will be made available upon request to the first author, g.alnahdi@psau.edu.sa.

## Ethics Statement

This study was reviewed and approved by the IRB Committee at Prince Sattam bin Abdulaziz University. Written informed consent was obtained from participants’ parents in this study.

## Author Contributions

GA designed the study and draft and analyzed the data. SS contribution was over all the paper and mainly in the introduction and discussion. Both authors contributed to the article and approved the submitted version.

## Conflict of Interest

The authors declare that the research was conducted in the absence of any commercial or financial relationships that could be construed as a potential conflict of interest.

## Figures and Tables

**FIGURE A1:**
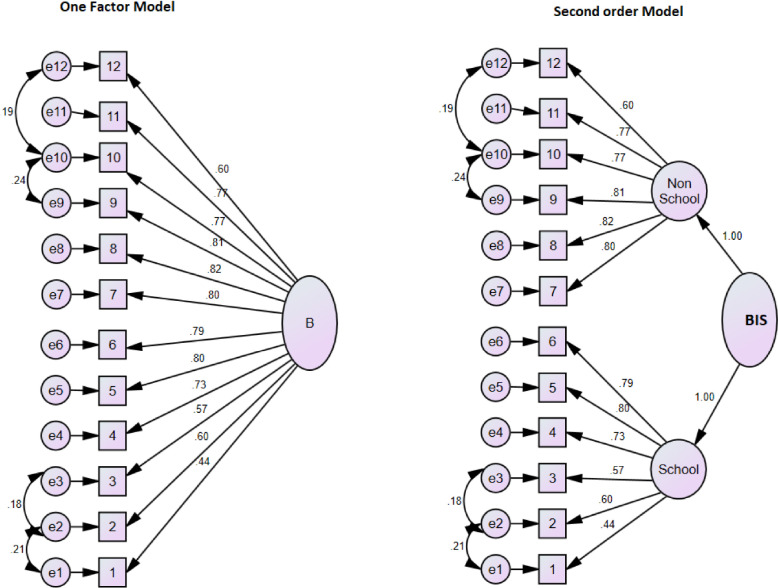
The CFA of the one-factor model and second-order CFA.
